# Attachment and the Development of Psychopathology: Introduction to the Special Issue

**DOI:** 10.3390/brainsci12020174

**Published:** 2022-01-28

**Authors:** Guy Bosmans, Jessica L. Borelli

**Affiliations:** 1 Clinical Psychology, Katholieke Universiteit Leuven, 3000 Leuven, Belgium; 2 Department of Psychological Science, University of California Irvine, Irvine, CA 92617, USA; jessica.borelli@uci.edu

When Bowlby [1] developed his attachment theory, he aimed to better understand the children and families he encountered in his clinical practice and to stimulate the development of effective treatments. Supporting the clinical relevance of his work, decennia of attachment research have shown a robust association between attachment and the development of psychopathology [2]. Therefore, it should not bear surprise that attachment theory has become one of the most influential theories in developmental psychology [3] and an important model that many clinicians rely upon to work with families and clients referred for mental health care.

According to attachment theory, children are born with a biological system that drives them towards proximity and support-seeking behavior when exposed to distress [1]. When parents respond in a sensitive and supportive way to their child’s distress, children develop internal working models of the parent as available for support and themselves as being worthy to be taken care for (called Internal Working Models of the self and others), and children develop a cognitive script about how to elicit care and how care-related interactions from others (called a Secure Base Script; [4]). These children are considered securely attached. When parents are less consistently available for support or when they are unavailable, children develop insecure attachment Internal Working Models and their knowledge of the secure base script is less developed. When distressed, these children show an enhanced focus on their attachment figures and display higher anxiety and stress about possible rejection or absent care by the parent (called ambivalent, preoccupied, or anxious attachment) or they start minimizing the importance of attachment figure and avoiding support seeking (called resistant, dismissing, or avoidant attachment). Many meta-analyses, including the ones in the current Special Issue (Dagan et al., this issue) demonstrate that insecurely attached children and adults are more at risk to develop psychopathology.

Thus, attachment is not only a visible marker linked with the development of psychopathology, it is also a developmental factor about which the main causal factor is assumed to be clear and tangible: parenting behavior [5]. The added value of a clear and tangible causal factor is that this allows designing interventions aimed at stimulating secure attachment development [6]. For these reasons, attachment theory has entered the jargon of every professional caregiver providing (mental) health care, child welfare, pediatric, psychiatric, and educational care [7]. Nevertheless, there are surprisingly much more ongoing debates regarding the role of attachment in psychopathology amongst researchers than the theory’s broad dissemination would suggest. The contributions to the current Special Issue provide new leads to consider (a) insecure attachment as a marker of psychopathology, (b) the mechanisms through which insecure attachment and the development of psychopathology are linked, and (c) the translation and implications of attachment theory for clinical practice.

**(a) Insecure Attachment as a Marker of Psychopathology**

The robust association between insecure attachment and psychopathology resulted in an overly negative appreciation of insecure attachment. It gives the impression that insecure attachment is a symptom of psychopathology. Nevertheless, this is clearly not the case. Bowlby himself argued that insecure attachment is an adaptive response to a suboptimal caregiving environment; in other words, the insecure child is a sane individual in a complex world. Additionally, in the work of Dagan et al. (this issue), effect sizes of the attachment-psychopathology association are modest, suggesting that a substantial number of insecurely attached individuals develop no symptoms of psychopathology. Research estimates that approximately 40% of the population is insecurely attached [8] which means that there is a substantially higher number of individuals who are insecurely attached than individuals who develop psychopathology. However, the same study also showed that of individuals seeking mental health care, 73% are insecurely attached. Therefore, insecure attachment is a highly relevant issue in clinical samples.

This again presents itself in the work by Dekkers et al. (this issue), where children with ADHD were compared with typically developing children and where, again, the rate of insecure attachment was significantly higher in the ADHD children. Interestingly, in the latter study, within the ADHD group, attachment (in)security was not related to the severity of behavior problems in this group. One explanation that stood out of the discussion was the possibility that children with ADHD failed to learn from care-related interactions with their parents. This interpretation fits with the recently developed learning theory of attachment [9]. According to this theory, the attachment system and children’s inclination to seek parental support during distress is part of the inborn genetic make-up of all children. However, whether or not children develop (in)secure attachments to their caregivers depends on basic classical and operant learning processes following the logic of safety conditioning (see Figure 1). Specifically, if during distress a parent (a Conditional Stimulus) is frequently paired with comfort and support (an Unconditional Stimulus), this will automatically elicit endocrinological changes (e.g., cortisol decreases, oxytocin increases; an Unconditional Response) which activates secure attachment states or state trust [10]. Over different learning events, if secure attachment states are repeatedly activated, children gradually develop the expectation that they can rely on parents for support (the Conditional Response). Hence, if secure attachment states are consistently activated, this seems to be linked to more secure trait attachment, whereas if there is more secure state attachment instability, this seems to be linked with decreases in secure trait attachment (Verhees et al., this issue).

Consequently, all factors that facilitate or disturb learning processes are supposed to affect children’s attachment development. Hence, it was important to see in Dekkers’ study (the current issue) that ADHD children’s attachment development was not linked to their behavior problems nor to parents’ expressed emotions. This suggests that the ADHD itself created problems to learn from interactions with parents, a finding that is in keeping with a recent cortisol study showing that the attachment development of children with higher levels of cortisol reactivity to stress is less linked to parents’ ability to provide support during distress [11]. Taken together, the current Special Issue confirmed first that not all insecurely attached children develop psychopathology (Dagan and Dekkers, this issue). Second, Dekkers et al. (this issue) showed that children with psychopathology are more at risk to develop insecure attachment. Third, adding a layer of complexity to the attachment-psychopathology link, Dekkers et al. (this issue)’s results suggest that some forms of psychopathology might be characterized by specific deficits that could hamper secure attachment development. More research is needed to further flesh out that last layer.

**(b) The Mechanisms Linking Insecure Attachment to the Development of Psychopathology**

Some 10 years ago, attachment researchers’ attention was drawn to the mechanism question [12], wondering what explains the link between insecure attachment and the development of psychopathology. A recently proposed model suggests that symptoms of psychopathology are the result of dynamic parent–child interactions that set off insecure cycles of interaction [13]; Figure 2. Insecure cycles are activated when children display distress followed by miscommunication, which means that children have the impression that parents do not respond sensitively to their distress. These miscommunications activate memories of past learning events during which children felt rejected or not supported. These activated insecure attachment states bias their processing of the attachment-relevant information in their environment due to which they will interpret their attachment environment as even more rejecting and which activates (fears for) negative emotions against which children will try to protect themselves. The more anxious or more avoidant self-defensive strategies they will then rely upon will eventually result in behavior that is in fact a distorted signaling of underlying attachment-related needs and fears. These behaviors trigger in the parent the fear of being an inadequate parent, unable to support their children’s development, or feelings of being unloved by the child. As a result, parents sometimes merely see the child’s distorted behavior and act on the appraisal that to save the child and their relationship with the child, the child’s (“mis”)behavior needs to be stopped. Therefore, parents use the (anxious or avoidant) self-defensive strategies they developed as a child to stop these behaviors. These are strategies parents rely upon to cope with their own fears of rejection and lack of support they feel now as a parent. These strategies eventually lead to behavior aimed to stop the pain caused by the child’s behaviors, but this behavior is perceived as “insensitivity” as it ignores the child’s underlying attachment needs and fears. Therefore, what once was a good coping when parents were children themselves, now interferes with their desire to be good parents for their own children. This activates the insecure cycle again in the child and can eventually lead to children displaying behaviors that are considered pathological.

The current Special Issue’s contributions fit nicely within the insecure cycle model. First, Verhees et al.’s study (this issue) showed support for the insecure cycles assumption that state attachment attachment variability can be linked to the development of psychopathology over time. Given that state variability reflects more instability in the care children experience, this suggests the occurrence of more insecure cycles and over time this translates to more distorted behavior that results from trying to deal with stress and the feared absence of care and that translates to psychopathology symptoms.

Second, results further support that the link between insecure attachment expectations and psychopathology runs through the strategies insecurely attached children rely on to cope with negative emotions. Two contributions (Iwanski et al., this issue; Tironi et al., this issue) supported the link between attachment and emotion regulation strategies. There is a growing body of literature on the role of emotion regulation as a mechanism explaining the link between insecure attachment and the development of psychopathology. Both Iwanski and Tironi’s contributions show how robust this link is over samples, over measurement methodology, and over time. Replication is key to move our field forward, and at least for that reason both contributions are highly valuable. However, both also significantly contribute to the literature. Iwanski’s study evaluated the relative contributions of attachment to mother and father and found cumulative effects, with children being insecurely attached to both parents showing more maladaptive emotion regulation strategies. This study is innovative as it accounts for the fact that child development is not merely affected by one parent, but by both parents and that this introduces a level of complexity that adds significantly to understanding why attachment is linked to the development of psychopathology. Tironi’s study is important as it accounts for potential biological mechanisms underlying emotion regulation responses to psychopathology. Their findings preliminarily suggest that attachment avoidance and anxiety interact with different physiological systems to predict risk for psychopathology; specifically, attachment avoidance is more likely to interact with respiratory sinus arrythmia (an index of parasympathetic nervous system response) to predict stress or psychopathology, whereas attachment anxiety is more likely to interact with electrodermal activation (an index of sympathetic nervous system response) to predict stress or psychopathology. An exciting area of future inquiry will be to examine the significance of these different pathways between different subtypes of attachment insecurity and different branches of the autonomic nervous system.

Third, the contribution of Bastin et al. (this issue) shows how children who are insecurely attached develop alternative behaviors driven by their self-defensive strategies. Bastin found that anxiously attached girls are more likely to engage in co-rumination with friends as they grow older. This may mean that some of them shift away from their parents and reduce their attempts to elicit care for their distress. Unfortunately, they are more at risk to engage in conversations with friends that emphasize their negative experiences such that they ruminate about it together. These adolescent girls may be replicating in their friendships an interactional pattern that began in their relationships with their caregiver(s)–whereas with their caregiver(s), with whom they were anxiously attached, the nature of their attachment involved feeling close and connected by virtue of negative emotion (anxiety, fear, need), their friendships now involve a dynamic in which the connection is forged through shared negativity. Future research on the insecure cycle could look into how this affects the parent–child interaction in co-ruminating adolescents. One possibility is that these adolescents feel supported by their friends in their anger and frustration about their parents and that this stimulates their sense of entitlement to stand up against parents in order to demand they care they feel they deserve but that they feel deprived from. Likely, such behavior activates parents’ needs to limit such behavior or activates parental behavior that endorses such behavior.

Finally, Dagan et al. (this issue) compellingly showed that the links between different self-defensive attachment strategies and psychopathology shifts with increasing age. Although the authors rightfully warn that this might just reflect methodological changes in how attachment and the symptoms of psychopathology are measured in different age-groups, the results could also indicate that a developmental shift occurs. This would mean that in adulthood the function of these self-defensive strategies shift. This is an intriguing finding that calls for replication and for new mechanism research. It raises the question why these functions shift. Dagan et al.’s (this issue) mis/match hypothesis suggests that the shift in function goes hand in hand with developmental shifts in orientation towards attachment figures. This is an interesting path to further pursue and could help to understand the function of depressive symptoms across the lifespan.

**(c) The Translation and Implications of Attachment Theory for Clinical Practice**

Finally, two contributions target intervention-related issues. First, Aafjes-Van Doorn et al.,’s study (this issue) adds to a recent number of studies showing that insecure attachment can have a negative impact on treatment outcomes [14]. The current study is specifically compelling for two reasons. First, they studied patients that were already in treatment before they were exposed to a serious stressor (i.e., COVID-19 and lockdowns) showing that this exposure did not dramatically add to these patients’ problems if they were more securely attached. This is important, as it suggests that the patients’ trust in the availability of support is a critical element for therapy to be successful. It has been argued that for these patients, restoring attachment ruptures might be a critical step necessary for (evidence-based) treatments to be successful [6].

In addition, the study showed that when insecurely attached patients were able to forge a good therapeutic relationship with their therapist, this acted as a buffer against the negative COVID-19 effect. This is hopeful, as it suggests that strategies should exist to build trust in relationships, even if patients have a learning history of attachment ruptures. The idea that attachment ruptures can be repaired fits with the learning theory of attachment that proposes that when the CS_parent_-UCS_comfort_ contingency improves, the CR_trait_trust_ will increase [15]. The challenge will be to bypass the biases in insecurely attached individuals’ processing of attachment information that are part of the enduring insecure cycles [16]. One way to achieve this is by exposing patients to their most intense negative emotions linked to their negative memories of past rejection and disappointment in their primary caregivers. If caregivers (including therapists) can respond to this pain in an emotion coaching way, allowing patients to share their deepest emotions and by acknowledging their pain, this can serve as a corrective attachment experience that stimulates trust [15].

Although therapeutic research suggests that this is a promising avenue [17], Newman-Taylor et al. (this issue) point at a second promising strategy. Specifically, they showed that priming secure attachment memories can also reduce psychopathology symptoms and increase support seeking intentions. This finding fits with the learning theory of attachment that builds on learning research showing that both positive and negative memories are stored in the brain but that the context determines to which memories individuals can have access (also demonstrated for attachment: [16]). Thus, priming can help to reactivate the more positive memories about interactions with attachment figures, which activates secure attachment states, and after repeated priming can consolidate in more trait trust and decreased symptoms. This work is consistent with the findings of other attachment-based interventions, such as relational savoring [18], which have as their goal helping clients recall memories of positive relational experiences (ideally, times when they provided or received sensitive care to/from an attachment figure) and engaging in deep emotional and cognitive processing of these memories. In line with attachment priming studies, relational savoring increases positive emotion, relationship satisfaction, and reflective functioning, while reducing psychopathology in parents and youth [18,19,20,21]. It will be interesting to see in future research whether attachment-based therapies focusing on creating corrective attachment experiences can be boosted using such more positive and stimulating intervention strategies as secure attachment priming and savoring.

## Conclusions

The true state of the science is far more complex than even Bowlby imagined, and we owe it to tomorrow’s children to develop and test theoretical models of the interplay between attachment and psychopathology that honor this complexity. The papers in this issue take us further in this quest to understand the true diversity of paths toward mental health present in the world and can stimulate more research to advance our knowledge.

## Figures and Tables

**Figure 1:**
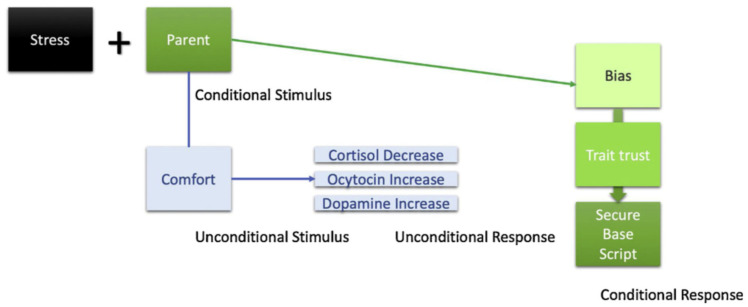
Safety Conditioning in Attachment Development.

**Figure 2:**
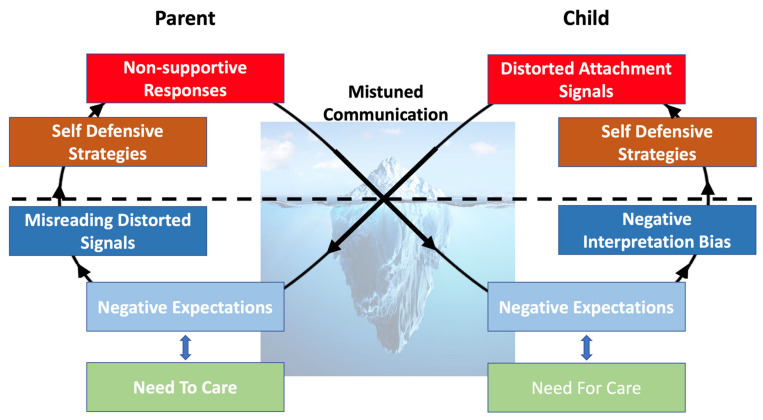
The Insecure Cycle.

## Data Availability

Not applicable.
